# Racial and Ethnic Differences in the Management of Atrial Fibrillation

**DOI:** 10.1016/j.cjco.2021.09.004

**Published:** 2021-09-13

**Authors:** Kamala P. Tamirisa, Sana M. Al-Khatib, Sanghamitra Mohanty, Janet K. Han, Andrea Natale, Dhiraj Gupta, Andrea M. Russo, Amin Al-Ahmad, Anne M. Gillis, Kevin L. Thomas

**Affiliations:** a Texas Cardiac Arrhythmia Institute, Austin/Dallas, Texas, USA; b Division of Cardiology, Duke University Medical Centre, Durham, North Carolina, USA; c Division of Cardiology, Veterans Affairs (VA) Greater Los Angeles Healthcare System, Los Angeles, California, USA; d University of California Los Angeles School of Medicine, Los Angeles, California, USA; e Department of Cardiology, University of Liverpool, London, United Kingdom; f Division of Cardiology, Cooper University Hospital, Camden, New Jersey, USA; g Department of Cardiac Sciences, University of Calgary, Calgary, Alberta, Canada

## Abstract

Atrial fibrillation (AF) is the most common clinical arrhythmia, and it results in adverse outcomes and increased healthcare costs. Racial and ethnic differences in AF management, although recognized, are poorly understood. This review summarizes racial differences in AF epidemiology, genetics, clinical presentation, and management. In addition, it highlights the underrepresentation of racial and ethnic populations in AF clinical trials, especially trials focused on stroke prevention. Specific strategies are proposed for future research and initiatives that have potential to eliminate racial and ethnic differences in the care of patients with AF. Addressing racial and ethnic disparities in healthcare access, enrollment in clinical trials, resource allocation, prevention, and management will likely narrow the gaps in the care and outcomes of racial and ethnic minorities suffering from AF.

## Graphical abstract

**Figure:**
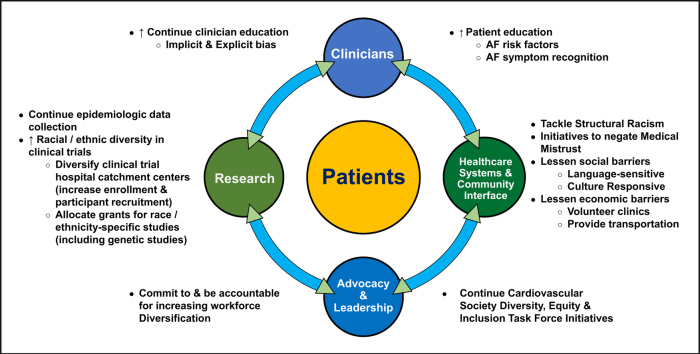

## Résumé

La fibrillation auriculaire (FA) est la forme clinique d’arythmie la plus fréquente, et elle entraîne des résultats défavorables ainsi qu’une augmentation du coût des soins de santé. Les différences liées à la race et à l’origine ethnique qui existent dans la prise en charge de la FA, bien que reconnues, sont mal comprises. Le présent article de synthèse résume les différences liées à la race observées sur le plan de l’épidémiologie, de la génétique, du tableau clinique et de la prise en charge de la FA. En outre, il met en lumière la sous-représentation de groupes raciaux et ethniques dans les études cliniques sur la FA, en particulier celles axées sur la prévention des accidents vasculaires cérébraux. Des stratégies *ad hoc* sont proposées pour que la recherche et les initiatives futures favorisent l’éradication des différences liées à la race et à l’origine ethnique dans les soins prodigués aux patients atteints de FA. Le fait de s’attaquer aux disparités liées à la race et à l’origine ethnique qui touchent l’accès aux soins de santé, l’inscription aux essais cliniques, l’allocation des ressources, la prévention et la prise en charge des patients permettra probablement de réduire les lacunes en matière de soins et de résultats chez les personnes atteintes de FA issues de minorités raciales et ethniques.

Atrial fibrillation (AF) is the most prevalent cardiac arrhythmia worldwide, estimated to affect 37.6 million people globally, and projected to increase by > 60% by the year 2050.^1^ Currently, ∼1 million people in Canada and 5.2 million in the US have AF, with the number in the US predicted to increase to 12.1 million by the year 2030.^2^^,^^3^ In Asia, the overall AF prevalence is ∼1%, which is less than that in Western countries. However, due to the overall size of the population in Asia, AF is estimated to affect 60-70 million Asian people by the year 2050.^2^^,^^4^ In Africa, the true prevalence of AF is underreported. Small cross-sectional studies reveal a prevalence of 0.7% to 5.5%, with some estimating that by the year 2050, the prevalence of AF in Africa will be the highest in the world.^3^^,^^5^

From 1997 to 2017, the number of years of health lost from AF increased by 77%, correlating with increasing age and high socioeconomic–index countries.^1^ Large variations were noted in AF prevalence across various regions of the world grouped by income level (7-fold increase in middle- income and 11-fold increase in high-income countries, compared with around 1 fold increase in low-income countries, where AF might be overlooked or underreported).^6^

Population-based studies have consistently reported a lower AF burden among racial and ethnic minorities, despite their having a higher prevalence of traditional risk factors. Given this, race-specific risk factors that might contribute to AF should be better elucidated. In addition, AF management differences for various races and ethnicities have been reported; however, these data have not been systematically reviewed. The present paper reviews global differences based on race and ethnicity in AF epidemiology, genetics, clinical presentation and management, and factors that might contribute to these differences, including systemic biases and social determinants of health. We highlight possible strategies to address these differences in diverse racial and ethnic groups to ultimately help improve adverse outcomes in these patients.

## Epidemiologic Factors of AF in Relation to Race and Ethnicity: AF Risk

Current epidemiologic studies have shown that AF is more prevalent in White patients than in other racial and ethnic groups. A recent study using the national inpatient sample (NIS) showed that of 30 million patients with AF at hospital discharge, significantly more White than Black patients had AF (11.3% vs 4.6%, *P* < 0.001).^7^ This significant difference in AF held true across all age groups above 50 years, and it was most significant in those > 75 years old (31.4% White vs 18.2% Black patients, *P* < 0.01).^7^ Similarly, 2 separate large studies of patients with AF in California hospitals showed significantly lower odds of having AF for all non-White races/ethnicities, compared with White patients (0.53 for Black, 0.61 for Hispanic, and 0.68 for Asians patients, *P* < 0.01 for all; and 0.49 for Black, 0.58 for Hispanic, and 0.68 for Asian patients, *P* < 0.0001 for all).^8^

In the **M**ulti-**E**thnic **S**tudy of **A**therosclerosis (MESA), after adjusting for age and sex, the incidence of AF was 49%, 46%, and 65% lower for non-Hispanic Black (NHB), Hispanic, and Chinese patients, respectively, compared with non-Hispanic White (NHW) patients.^9^ Another study of 417,525 patients from the United Kingdom showed that South Asians had 71% lower odds of having AF, with an age-adjusted prevalence of 0.4% vs 2.4%, compared with White patients.^10^ The few studies that have been performed in South Asia show an AF prevalence of 0.1%-0.6%.^11^

Black individuals have a lower reported prevalence of AF compared with White individuals, despite having a higher prevalence of traditional AF risk factors; this disparity is termed the “AF paradox.” 12, 13, 14, 15 In the **Re**asons for **G**eographic **a**nd **R**acial **D**ifferences in **S**troke (REGARDS) prospective cohort study of nearly 14,000 patients followed for 9.4 years, Black individuals had a 0.46 relative risk of developing AF compared with their White counterparts, while having significantly more diabetes, hypertension, and obesity.^16^ This paradox has also been noted in South Asians, who have higher age-adjusted rates of diabetes, hypertension, and coronary artery disease compared with Whites in the United Kingdom, but a very low rate of AF.^10^^,^^17^ Several theories have been proposed to explain this AF paradox, including differences in healthcare access, survival bias with longer life expectancy in NHWs leading to an increased risk of AF, and possible differences in symptom recognition and perception.^8^^,^^18^ Left atrial (LA) size and volume have been shown to differ across races, with larger LA size observed in White compared with East Asian, South Asian, and some Black populations.^19^ Larger LA size/volume confers a higher AF risk, whereas smaller LA geometry confers a protective effect from AF.^20^, ^21^, ^22^ Most recently, Black patients have been found to have a unique extracellular matrix biomarker response that could be associated with attenuation of hypertensive atrial remodelling.^22^ However, a recent study from a MESA cohort showed that although the prevalence of clinically detected AF was, again, lower in Black patients than White patients, the use of 14-day ambulatory electrocardiogram (ECG) patch monitoring showed no difference in AF prevalence among races/ethnicities.^23^

## Is There Ascertainment Bias in AF Detection by Race?

Some investigators have raised the possibility of an ascertainment bias in AF detection by race, owing to challenges in accessing health care.^18^ For example, a study screening for AF using mobile ECG devices found a 3-fold higher AF prevalence in South Asian patients than that in previous reports, suggesting ascertainment bias.^24^ Similarly, a substudy of 1556 participants of the MESA cohort looked at clinically detected AF vs AF detected by 14-day ambulatory ECG monitoring. Although the prevalence of clinically detected AF was higher in White than in Black, Hispanic, or Chinese patients after 14.4 years of follow-up, the proportion of individuals with monitor-detected AF over 14 days was similar among the 4 groups.^23^ However, other studies, either using 14-day ambulatory ECG monitoring or following patients with implantable cardiac devices to detect subclinical AF over longer periods of time, do not corroborate these observations.^25^, ^26^, ^27^, ^28^ The characteristics and outcomes of these 5 different studies are summarized in Table 1. The differences in outcomes of these studies stress the need to adopt better monitoring in more-diverse samples of patients to gain further clarity on the role of ascertainment bias in the AF paradox.

**Table 1:** Summary of characteristics and outcomes of 5 studies

Study	Study design	N	Mean duration of follow-up	Main results	Comments
Heckbert et al.,^23^ 2020	Cross-sectional analysis of a community-based cohort	1556	14.4 y	The prevalence of clinically detected AF was 11.3% in White, 6.6% in Black, 7.8% in Hispanic, and 9.9% in Chinese patients. Monitor-detected AF using a 14-day ambulatory ECG monitor was similar in the 4 race/ethnicity groups: 7.1%, 6.4%, 6.9%, and 5.2%, respectively	Lower prevalence of cardiovascular disease
Chen et al.,^27^ 2019	Retrospective cohort of Medicare beneficiaries with implanted devices. (study used inpatient & outpatient claims from 2009 to 2015).	47,417	2.3 y	Annual incidence of AF /atrial flutter was 12.2 per 100 person-years in Black patients, and 17.6 per 100 person-years among non-Black patients. Adjusted results showed Black beneficiaries had a lower risk of AF /atrial flutter than non-Black patients (hazard ratio, 0.75; 95% CI , 0.70–0.80)	Miscoding and misclassification errors are possible
Rooney et al.,^25^ 2019	Cross-sectional analysis of a community-based cohort. (participants used a leadless, ambulatory ECG monitor Zio XT [iRhythm Technologies, San Francisco, CA] Patch for up to 2 weeks)	2616	4 wk	The prevalence of subclinical AF was 3.3% in White men, 2.5% in White women, 2.1% in Black men, and 1.6% in Black women.	Small numbers of Black men (214) and Black women (469). Short follow-up.
Kamel et al.,^28^ 2016	Retrospective cohort study using administrative claims data in California, Florida, and New York (either 2005 or 2006 to 2010 or 2011)	10,393 Black and 91,380 White patients with no known AF or atrial flutter before or during the initial encounter for pacemaker implantation	3.7 y	Black patients had a significantly lower risk of AF (21.4%; 95% CI 19.8–23.2) than White patients (25.5%; 95% CI 24.9–26.0). Adjusted data showed that Black patients had a lower hazard of AF (hazard ratio 0.91; 95% CI 0.86–0.96) and a higher hazard of atrial flutter (hazard ratio 1.29; 95% CI 1.11–1.49)	Miscoding and misclassification errors are possible
Lau et al.,^26^ 2013	Secondary analysis of a prospective multicentre cohort study	2,580	2.5 y	All 3 non-White race groups had a lower incidence of AF (8.3%, 10.1%, and 9.5% vs 18.0%, respectively, for AF > 6 min, *P* < 0.006). Adjusted data showed that Chinese patients had a lower incidence of AF > 6 minutes (*P* < 0.007), and Japanese and Black patients had a lower incidence of AF > 6 h (*P* < 0.04 and *P* = 0.057, respectively).	Small number of non-White patients; 73 Black patients, 89 Chinese patients, and 105 Japanese patients

AF, atrial fibrillation; CI, confidence interval; ECG, electrocardiogram.

## Genetics of AF

Genome-wide association studies over the past 15 years, performed largely in those of European descent, have identified 9 loci associated with AF risk.^29^ In order to delineate the role of genetic factors in AF, Marcus et al.^30^ studied White and Black patients of European Ancestry from the Cardiovascular Health Study (CHS) and **A**therosclerosis **R**isk **i**n **C**ommunities (ARIC) study. They found that for every 10% increase in European ancestry, there was a 13% increased risk of AF, even after adjusting for potential confounders.^30^ Further work on 18,919 individuals of European ancestry found an up to 67% increased risk of new-onset AF for those in the highest quartile of genetic risk scores compared with those in the lowest quartile, exceeding the effects of traditional AF risk factors.^31^ Multiethnic studies of AF genetics in underrepresented populations are beginning to be conducted.^32^ Genome-wide admixture analysis and candidate single nucleotide polymorphism (SNP) study of the ARIC, the CHS, and the Health, Aging, and Body Composition (Health ABC) cohorts found that the minor protective allele of the AF SNP rs10824026 was more common among Black than White patients. However, as no novel genome-wide significant genetic variant was found in a meta-analysis, the authors felt that this difference was unlikely to fully account for the AF paradox.^33^ In a study of Han Chinese individuals that analyzed 6 SNPs associated with AF in those of European ancestry, SNP rs3807989 in the *CAV1* gene on chromosome 7q31 was found to be significantly associated with a decreased risk of AF (adjusted odds ratio = 0.75; 95% CI: 0.63-0.89, *P* = 0.001).^34^ In Hispanic patients, the presence of SNP rs10033464 at chromosome 4q25 conferred a 2.3-fold increase in the risk of AF after multiple risk-factor adjustment.^35^ Recent work from the same group sequencing 60 candidate genes from Black and Hispanic/Latino people with early-onset AF was able to identify likely pathogenic variants in a small number of probands.^36^ Black and Hispanic patients with early-onset AF have been found to have higher odds than White patients of having a first-degree family member with AF, supporting genetic predispositions that should be further explored.^37^

Whether environmental or genetic, there appear to be ethnicity-specific factors that either protect Black patients from AF or make White patients more prone to AF.^38^ Although genetics cannot fully account for the differential AF risk across races, its role with or without the contribution of ascertainment bias in AF detection by race warrants further investigation, as it could provide important insight into the AF paradox in the Black population. However, available data suggest that environmental and genetic factors contribute to the paradoxical race-related risk of AF among White and Black individuals.^33^

## Differences Relating to Race and Ethnicity in Symptoms and Quality of Life

**Figure 1:**
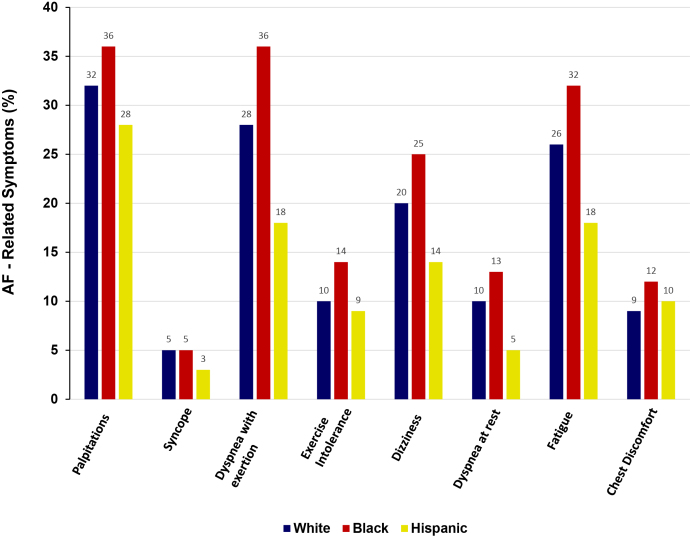
Symptoms of AF. AF, atrial fibrillation.

There appear to be differences in symptoms attributable to AF that are based on race and sex.^18^^,^^39^ The **O**utcomes **R**egistry for **B**etter **I**nformed **T**reatment of **A**trial **F**ibrillation (ORBIT-AF), a multicentre, prospective registry of outpatients with incident or prevalent AF, examined racial and ethnic differences in quality of life, treatment, and outcomes associated with AF.^39^ Compared with White individuals or those of Hispanic ethnicity, Black patients reported more severe symptoms associated with AF. Black patients were more likely to report palpitations, dyspnea on exertion, decreased exercise tolerance, dizziness, dyspnea at rest, fatigue, and chest discomfort compared with White and Hispanic patients (Fig. 1). Based on the European Heart Rhythm Association AF symptom scores reported at baseline, Black patients reported more severe or disabling symptoms (20.4%), compared with White (16.4%) and Hispanic patients (8.5%). Furthermore, Black patients reported lower overall quality-of-life scores within 2 years of follow-up.^39^

## Are There Delays in Presentation and Diagnosis of AF?

Specific data on racial disparities related to delays in seeking medical attention for symptoms related to AF, and investigations to diagnose AF, are lacking. However, sex- and race-based differences in access to more advanced interventions for cardiovascular diseases, including coronary artery disease and heart failure, are well documented.^40^ Not all of these differences can be explained by a lack of either health insurance coverage or access to health care. Factors such as socioeconomic status and lack of supplemental insurance coverage, as well as documented sex- and race-based differences in preferences related to medical care, might contribute to delays in diagnosing AF.^41^ Lack of health literacy may limit patient engagement and impact decisions for treatment of AF. Non-White patients and those with a lower education level have been shown to be less likely to receive care from a cardiologist for AF.^42^ Some data report that women and Hispanic patients are less likely to be referred to an electrophysiologist or a tertiary-care centre specializing in AF management.^43^

In a US biracial study investigating racial disparities in stroke risk among individuals confirmed to have AF by ECG, Black patients were approximately one third as likely to be aware that they had AF as White patients.^44^

## Racial and Ethnic Differences in AF Outcomes

Studies have found racial and ethnic differences in the outcomes of AF. Data from the prospective ARIC study showed that although both Black and White patients with AF had higher risk of stroke, heart failure, coronary artery disease, and death than those without AF, Black patients with AF had a 1.5 to 2 times higher risk for each outcome than White patients with AF.^45^ A Medicare claims database study examining > 500,000 patients over 65 years of age with AF showed that Black and Hispanic patients had a higher unadjusted risk of death (46% and 11%, *P* < 0.001 for both) and stroke (66% and 21%, *P* < 0.001 for both) than White patients. Adjusting for demographics and comorbidities eliminated the higher risk of death, but stroke risk remained significantly higher in Black and Hispanic patients (46% and 11%).^46^ In a smaller study of 236 patients, Mexican American patients with AF had a 2-fold higher risk of recurrent stroke and greater stroke severity than a comparator group of NHW patients with AF; however, there was no difference in all-cause mortality.^47^ Lastly, in a large cohort (**N**ational **C**ardiovascular **D**ata **R**egistry [NCDR] **P**ractice **Inn**ovation **a**nd **Cl**inical **E**xcellence [PINNACLE] Registry) study of North-American Asian patients and White patients with AF, there were no significant race-based differences in stroke, life-threatening bleeding, or all-cause mortality.^48^ However, this lower risk does not necessarily translate to non–North American Asians.^2^ AF-associated stroke prevalence across Asia is reported to be as low as 1.3% to as high as 15.4%, depending upon the geographic area studied and the availability of oral anticoagulation.^2^ Both ischemic and hemorrhagic stroke incidences were higher for NHB and Asian patients in a subanalysis of the **R**ivaroxaban **O**nce Daily Oral Direct Factor Xa Inhibition **C**ompared with Vitamin **K** Antagonism for Prevention of Stroke and **E**mbolism **T**rial in **A**trial **F**ibrillation (ROCKET AF).^49^^,^^50^ In addition, both ischemic and nonischemic stroke incidences were higher for East Asian vs non–East Asian individuals in the **R**andomized **E**valuation of **L**ong-Term Anticoagulant Therap**y** (RE-LY) and the **A**pixaban for **R**eduction **i**n **St**roke and **O**ther **T**hromboembolic **E**vents in Atrial Fibrillation (ARISTOTLE) trials, indicating a higher risk of both types of stroke with anticoagulation in some Asian patients.^2^ Lastly, intracranial hemorrhage has also been shown to be significantly higher in non-White vs White patients with AF on anticoagulation. In a large retrospective multiethnic cohort of > 18,000 AF patients on warfarin without previous stroke, hazard ratios for intracranial hemorrhage were 4.06 for Asians, 2.06 for Hispanics, and 2.04 for Blacks.^51^

## Disparities in Treatment of AF

### Anticoagulation

Stroke prevention is one of the most important therapeutic goals in AF management. Several groups have highlighted major racial differences in care in relation to stroke prevention in AF. Historical studies and Medicare data have shown that NHB and Hispanic patients with AF were significantly less likely to be prescribed vitamin K antagonists than were other ethnic groups, despite having higher stroke-risk scores, and they were also frequently lost to international normalized ratio (INR) monitoring.^39^^,^^44^^,^^49^^,^^52^, ^53^, ^54^ Similarly, Black race was associated with a significantly decreased time in therapeutic range, compared with NHW race, even after adjusting for medication compliance and monitoring frequency.^55^ In a mixed cohort in the United Kingdom, the quality of INR control was shown to be inferior in African Caribbean and South Asian patients compared with White patients, despite similar INR testing frequency.^56^ Non-White ethnicity remained the strongest independent predictor of poor therapeutic range.^56^ Among 11,575 newly diagnosed AF patients with a CHA_2_DS_2_VASc (**C**ongestive Heart Failure, **H**ypertension, **A**ge ≥ 75 years, **D**iabetes Mellitus, **S**troke, **V**ascular Disease, **A**ge 65 to 74 Years, **S**ex **C**ategory) score of 2 or more, after adjusting for multiple factors, including income and insurance status, the odds of receiving any oral anticoagulant was 0.69 (95% confidence interval [CI]: 0.58-0.83) in Black patients, 0.74 (0.53-1.90) in Hispanic patients, and 0.75 (0.58-0.95) in Asian patients, compared with White patients.^57^ The odds of receiving direct oral anticoagulants (DOACs) among those who were prescribed oral anticoagulants were lower for Black and Hispanic patients.^57^ Among those with prior gastrointestinal bleeding, White patients were nearly 5-fold more likely to be discharged on an oral anticoagulant than were non-White patients, independent of income.^58^

Lower use of DOACs in Black and Hispanic patients has been reported in other studies. In the ORBIT-AF study, Black race, and Hispanic ethnicity, relative to White race, were associated with lower odds of being switched from warfarin to a DOAC.^59^ A Veteran Affairs study showed that NHW patients were more likely than NHB patients and those of other races or Hispanic ethnicity to be switched from warfarin to dabigatran in the setting of labile INR.^60^ The FLorida Puerto Rico Atrial Fibrillation Stroke Study Stroke study found that Black patients had the lowest rate of DOAC initiation at discharge.^61^ Data from the Get with The Guidelines–Stroke program, showed that Black, Asian, and Hispanic patients had lower odds of DOAC prescription, relative to NHW patients.^62^ Similar results were seen among Medicare beneficiaries.^63^

### Left atrial appendage occlusion

Left atrial appendage occlusion (LAAO) has emerged as a promising alternative stroke-risk–reduction strategy in patients who are deemed unsuitable for long-term oral anticoagulant therapy.

There has been a lack of inclusion of diverse populations in large trials involving percutaneous LAAO; 91%-94% of participants in the Percutaneous LAAO for WATCHMAN LAA System for Embolic **Protect**ion in Patients With **A**trial **F**ibrillation (PROTECT-AF) and the **P**rospective **R**andomized **Eva**luation of the WATCHMAN LAA Closure Device **i**n Patients With Atrial Fibrillation vs **L**ong-Term Warfarin Therapy (PREVAIL) study were White.^64^^,^^65^ In the NCDR LAAO registry, with over 38,000 Watchman procedures conducted between 2016 and 2018, only 7.4% patients were non-White.^66^

Most data on racial, ethnic, and socioeconomic disparity in LAAO have emerged from the NIS. Three studies using NIS data on hospitalization for the LAAO procedure reported underrepresentation, greater complication rate, longer hospital stay, and higher discharge complexity in the non-White population receiving LAAO, compared with White patients.^67^, ^68^, ^69^ Lower socioeconomic status was not associated with a higher complication rate. 67, 68, 69 In another analysis using the same NIS data, only 12.4% of non-White patients were reported to have undergone the LAAO procedure in the US.^70^ Higher odds of mortality and other adverse outcomes were reported in one study among non-White patients undergoing the LAAO procedure, compared with odds for White patients.^71^ One hypothesis for why the complication rate is higher in non-White patients is that these procedures are performed in low-volume centres that may have worse outcomes. Caution must be exercised in drawing the inference that individuals from diverse racial and ethnic backgrounds are more prone to complications or in using this as a reason not to consider LAAO. Rather, these results call for continued investigation of reasons for racial and ethnic differences in adverse outcomes in AF.

A similar lack of diversity has been documented in the surgical LAAO. Patient data extracted from a large US administrative database showed underrepresentation (∼20%) of non-White patients.^72^ In another retrospective analysis of Medicare recipients with AF undergoing cardiac surgery, the majority (93%) of patients receiving surgical LAAO were White.^73^

### Rate control vs rhythm control

Historically, there has been a paucity of studies assessing rate- vs rhythm-control approaches among different racial and ethnic populations with AF. Examination of the ORBIT-AF registry demonstrated a low enrollment of Black (5%) and Hispanic (4%) patients.^39^ Over the past several years, the emergence of AF registries and the use of administrative datasets have provided the infrastructure to assess this issue. Studies utilizing these registries have demonstrated that Black and Hispanic patients are less likely than White patients to receive rhythm-control or catheter-ablation treatment, despite the presence of symptoms.^39^^,^^41^ A recent meta-analysis reported similar rates of atrioventricular node ablation but lower rates of AF ablation among Black patients, compared with White patients.^18^

In the **A**trial **F**ibrillation **F**ollow-Up **I**nvestigation of **R**hythm **M**anagement (AFFIRM) randomized clinical trial, 90.1% of patients were White, and only 6.6% and 3.3% of patients were Black and Hispanic, respectively. Black and Hispanic patients tended to have a higher prevalence of heart failure and a lower ejection fraction.^74^ Hispanic patients were more likely than White patients to be taking digoxin, but there was no difference in the use of beta-blockers or calcium-channel blockers between the 2 groups. The AFFIRM analysis did not compare achievement of adequate rate control among different races/ethnicities.^74^

### Rhythm control: pharmaco-therapy and catheter ablation

In an analysis of the ORBIT-AF registry, White patients, relative to Black and Hispanic patients, had undergone more cardioversions and catheter ablations for AF and were treated with more anti-arrhythmic therapies.^39^ Similarly, in a Medicare dataset, individuals identified as having Hispanic ethnicity were less likely to undergo catheter ablation, relative to individuals identified as being of White race.^40^ A sample study using hospital inpatient discharge and the ambulatory outpatient datasets from the Florida Agency for Healthcare Administration between the years 2006 and 2009 has shown that Black and Hispanic patients were less likely to undergo catheter ablation for AF than were White patients, with adjusted odds ratios of 0.67 (95% CI 0.60-0.75, *P* = 0.01) and 0.83 (95% CI 0.75-0.91, *P* = 0.01), respectively.^75^ A recent 2021 publication analyzed data from the Optum Clinformatics Data Mart, a deidentified database of administrative claims by members of a commercial insurance plan and Medicare Advantage plan that included patients with paroxysmal AF. The findings were consistent with those of prior studies; compared with White patients, Black patients were significantly less likely to be treated with rhythm control (adjusted odds ratio 0.89 [95% CI 0.83-0.94], *P* < 0.001), and those of Latino ethnicity were significantly less likely to undergo catheter ablation.^41^ Data from the PINNACLE/NCDR registry were utilized to assess AF management among American Indian and Alaskan Native patients. These patients were significantly less likely to be treated with rhythm-control strategies, compared with non-American Indian/Alaskan Native patients.^76^

Another single-centre study that included 792 patients showed that most patients who received catheter ablation for AF were White (93.3%), rather than Black (2.1%), Hispanic (0.1%), or Asian (0.9%).^77^ In a hospitalized cohort of AF patients, Naderi et al.^78^ reported the lowest rates of catheter ablation among Black women, a difference for that specific subgroup that has been noted in other invasive electrophysiology procedures.^79^^,^^80^

A noteworthy point is that randomized clinical trials and a meta-analysis of recent trials have shown that catheter ablation compared with antiarrhythmic drugs is associated with reductions in recurrence of atrial arrhythmias and hospitalizations, with no difference in major adverse events.^81^ Recent studies, namely the East AF Network (EAST AFNET) trial and the Early Aggressive Invasive Intervention for Atrial Fibrillation (EARLY-AF) trial, have suggested that early rhythm control of AF may be beneficial in many patients with AF.^82^^,^^83^ Current guidelines recommend catheter ablation as a treatment option for patients with heart failure with reduced ejection fraction and AF, as catheter ablation has been shown to decrease mortality and heart failure hospitalizations, as well as increase left ventricular ejection fraction in these patients.^81^^,^^84^ Given the higher heart failure burden in racial and ethnic minorities compared with that in White individuals, racial and ethnic minorities particularly may benefit from rhythm-control strategies.

## Social Determinants of Health

Many factors might contribute to the racial and ethnic disparities in AF management, including systemic biases, social determinants of health, trust in healthcare providers, health literacy, and perceived differences in response to therapy that may or may not be real.^85^ As previously mentioned, Black race and lower income were independently associated with lower use of rhythm control and, among patients receiving rhythm control, Hispanic ethnicity and lower income were independently associated with lower use of catheter ablation.^41^ The lower utilization of rhythm-control interventions in Black and Hispanic patients may equate to the delivery of lower-quality care, or conversely, this difference arguably may represent overutilization of rhythm-control interventions in White patients.

Private insurance and higher household income were also associated with greater likelihood of undergoing ablation in another study.^86^ In addition, patients with Medicare or Medicaid coverage, and uninsured patients, had lower rates of AF-ablation treatment compared with those with private insurance.^87^

Racial differences in outcomes for hospitalized patients with AF have been identified.^88^ In a study using the NIS of hospitalized patients with the principal diagnosis of AF, Black race was associated with an increased risk of death. This occurred particularly in hospitals with larger proportions of Black patients, compared to hospitals that had fewer Black patients, suggesting that Black patients may be more likely to be admitted to poorer-performing hospitals.^88^ Regional differences in stroke incidence in patients with AF have also been reported in the US, with the highest age- and sex-standardized stroke incidence occurring in the Middle Atlantic and East South-Central regions, and the lowest occurring in the West North-Central region.^89^

Improving awareness of the differences in the clinical community would potentially tackle systemic bias in clinical research and data interpretation.^90^ In this context, a noteworthy point is that health behaviour can be influenced by many factors. Education, cultural and religious preferences, acceptance of new drugs, devices, and procedures, as well as access to health care based on economic status play critical roles in health-related outcomes.^91^ Additionally, with an increasing number of people belonging to multiple racial and ethnic categories, interpretation of data based on race and ethnicity is challenging.^91^

Understanding these potential contributors to racial and ethnic disparities in AF management is clearly beyond the scope of administrative studies of commercially insured patients. Such understanding requires a deeper look into individual patients, the role of social factors, and interaction of patients with physicians.^85^ Heterogeneity within populations must be recognized so that care can be better customized. Use of the word “ethnicity” interchangeably with “race” by biomedical researchers hinders heterogeneity-based data analysis. Hispanic and Latino populations are diverse and come from different parts of the globe with differing ethnic backgrounds, including Puerto Rico, the Dominican Republic, Mexico, and other parts of South America, as well as Spain. Asians are not only East Asians but also South Asians. Given this range, data collection and classification should be more granular, using terms that allow for better scientific precision and communication.^90^

Understanding the dynamics of how social determinants of health affect adherence to treatment is vital before labeling patients as “noncompliant.” A set of skills and approaches, using the mnemonic “RESPECT” (*R*espect, *E*xplanatory Model, *S*how Context, *P*ower, *E*mpathy, *C*oncerns/Fears, *T*rust) to better understand a patient’s values and autonomy, has been suggested to help clinicians at all levels mitigate the social determinants of health barriers.^92^

## Possible Solutions to Help Narrow the Gaps

The National Healthcare and Quality Disparities report highlights pervasive racial and ethnic disparities in healthcare access, quality of healthcare delivery, and outcomes.^93^ As detailed in this article, these disparities have plagued all aspects of AF management. Important to note is that studies on disparities in AF care have been conducted mostly in the US, which begs for a global call to conduct studies in all regions of the world. Data specific to each region are instrumental to inform the development and customization of strategies most likely to be effective in that region. A multi-pronged approach is needed to address these disparities in AF care.

As mentioned earlier, existing clinical trials generating the evidence on efficacious and effective therapies for AF have not included racially and ethnically diverse populations. In a recent study, only 44% of randomized clinical trials on AF reported participant-level information on race/ethnicity.^94^ Black and Hispanic patients accounted for just 2% and 5.6% of trial participants, despite making up 13.4% and 18.3% of the US population in 2018.^94^ These findings document the striking lack of racial and ethnic diversity in AF trials that limits the generalizability of the results to the global population. Poor access to health care also contributes to under-recruitment in clinical trials, which in turn results in poor understanding of race-specific risk factors.^95^ As a recent example, a study examined one of the latest advancements in AF ablation technologies—pulsed field ablation,a non-thermal energy modality with several potential advantages over existing ablation modalities.^96^^,^^97^ The racial and ethnic distribution of the study population was not provided in the human safety and efficacy pulsed field ablation trials.^96^^,^^98^, ^99^, ^100^ Moreover, catheter ablation using the standard radio-frequency energy in the **P**rospective **R**eview of the Safety and **E**ffectiveness of the THERMOCOOL SMARTTOUCH SF **C**atheter **E**valuated for Treating Symptomatic **P**ersisten**t** AF (PRECEPT) trial was used less commonly for Black patients, but the technology was deemed to be equally effective across all racial cohorts.^101^^,^^102^

Underenrollment of diverse races and ethnicities into AF clinical trials and studies can be addressed in the following ways:

• The sample size of racial and ethnic groups in traditional and pragmatic clinical trials with granular data capture should be prespecified. Post-marketing studies will provide much needed data in unlocking the causal mechanisms associated with worse outcomes in these populations.
• Multicentre international or global trials should be designed with diversified hospital-catchment areas, including communities with diverse races, ethnicities, and socioeconomic strata. Such design is key to a better understanding of the barriers to underrepresentation in clinical trials.
• Practical considerations, including transportation and language barriers, should be taken into account. Hiring of diverse advocacy workers by the study administrators can help improve communication between recruiters and the minority population.^103^ If and when possible, remote follow-up and mobile-health technology should be utilized for patient monitoring, with provision of digital health technology to prevent worsening of the digital divide.

Data on AF collected by both administrative claims and registries are in need of improvement, as studies utilizing these methods to examine racial and ethnic differences in AF care can be fraught with limitations.^90^ The decision to pursue rhythm control can be complicated and should incorporate several important factors, such as symptoms, burden of AF, and –left ventricular ejection fraction, none of which are well captured in administrative claims data and many registries. Studies of differences in AF care should collect and report these data and should consider that many times a clear preference for one form of treatment over another is not obvious. In addition, administrative data lack reasons that differences in treatment exist. Appropriately designed registries can provide these data.^90^ The current use of “ethnicity” in biomedical research as a binary term (which is the current standard in the US) is flawed and needs to be revamped and standardized to separate race from ethnicity.

Insurance and socioeconomic barriers are important contributors to differences in AF care. Racial and ethnic minorities are less likely to be insured and to have primary care providers who play a key role in early recognition and prevention of AF.^47^ Moreover, there is a dearth of studies reporting on AF management in different races matched on ability to pay. When more data from single-payer health systems emerge in the near future, it will be important to examine racial and ethnic differences in AF management.

Moreover, the US population is projected to become even more racially and ethnically diverse in the next few decades.^47^ By 2060, Hispanic patients will make up 29% of the US population, up from 17% in 2014; individuals with Black race alone or in combination with one or more other races will make up nearly 18% of the total population, up from 14% in 2014.^12^ Racial disparities in healthcare outcomes persist, even after adjusting for socioeconomic factors.^104^ Although these issues are not easy to address, new health coverage plans, and customized clinical workflows, are important to enhance access to and quality of care received by underrepresented racial and ethnic minorities.^104^

As previously mentioned, cultural factors can affect the AF patient’s perception of illness and symptom reporting. This in turn may result in underdiagnosis and suboptimal care in a racially and ethnically diverse population of patients. Patient education has been shown to improve recognition of symptoms of heart disease in general.^105^ AF is a complex disease for patients, owing to its specialized terminology, symptom recognition, and complicated therapeutic options requiring shared decision-making.^106^ There is a critical need to understand whether integration of health literacy into the care of patients with AF improves management, including decision-making, treatment adherence, persistence, and outcomes. ^106^

Clinicians’ implicit biases add another confounding layer to the underlying apprehension and mistrust experienced by patients from underrepresented backgrounds and can contribute to differences in AF care. Implicit biases are pervasive among all, including physicians, administrators, allied professionals, and nurses.^107^ Repetitive and purposeful interventions on both individual and systemic levels are essential to move the medical community forward in this regard.^108^ Taking self-assessment tests and soliciting feedback are initial key steps toward addressing and mitigating individual-level bias.^109^ Including diversity and inclusion training as an integral part of the curriculum is essential for systematic, grassroots change.^110^ Racially diversifying the workforce improves healthcare quality and minority patients' utilization of health services, leads to more culturally responsive and patient-centred interaction, and enhances research with a broader range of racial/ethnic perspectives.^111^^,^^112^ Diversity among research investigators enhances involvement of underrepresented populations in research planning.^113^ Concerted and intentional efforts should be made to form diverse and inclusive study teams, develop methodological strategies backed by conceptual frameworks, and train investigators to avoid generalized hypotheses to answer race- and ethnicity-specific questions.^90^ Training future physicians on cultural humility and structural racism, equity-oriented healthcare initiatives, and advocacy for reformation is vital to maintain the momentum of change toward health equity for future generations. Without tackling structural racism from all angles, health inequities will persist. A concerted effort will help narrow the gaps in health care and beyond.^114^

Finally, professional societies and organizations play a pivotal role in addressing differences, as they are the public face and voice of cardiovascular professionals. The Heart Rhythm Society has paved the way with the formation of the Diversity, Equity and Inclusion Task Force, consisting of stakeholders and leaders who are committed to providing an all-inclusive environment, by striving for leaders who represent all aspects of diversity with the goal of narrowing gaps in healthcare outcomes for patients with not only AF, but also other electrophysiological disorders.^115^

## Conclusion

The world is at a critical crossroad regarding the relationship of race and ethnicity with healthcare outcomes. By providing comprehensive data on disparities in AF management and proposing potential strategies to address these disparities, it is hoped that this paper will create more momentum for future work in this area. It is high time for the medical community to seize the opportunity to test and implement strategies that reduce differences and improve the care and outcomes of all patients from underrepresented racial and ethnic backgrounds with AF worldwide.

## References

[1] Lippi G., Sanchis-Gomar F., Cervellin G. (2021). Global epidemiology of atrial fibrillation: an increasing epidemic and public health challenge. Int J Stroke Soc.

[2] Chiang C.-E., Okumura K., Zhang S. (2017). 2017 consensus of the Asia Pacific Heart Rhythm Society on stroke prevention in atrial fibrillation. J Arrhythmia.

[3] Stambler B.S., Ngunga L.M. (2015). Atrial fibrillation in Sub-Saharan Africa: epidemiology, unmet needs, and treatment options. Int J Gen Med.

[4] Wong K.C., Klimis H., Lowres N. (2020). Diagnostic accuracy of handheld electrocardiogram devices in detecting atrial fibrillation in adults in community versus hospital settings: a systematic review and meta-analysis. Heart.

[5] Rahman F., Kwan G.F., Benjamin E.J. (2014). Global epidemiology of atrial fibrillation. Nat Rev Cardiol.

[6] Joseph P.G., Healey J.S., Parminder R. (2021). Global variations in the prevalence, treatment, and impact of atrial fibrillation in a multi-national cohort of 153 152 middle-aged individuals. Cardiovasc Res.

[7] Kowlgi G.N., Gunda S., Padala S.K. (2020). Comparison of frequency of atrial fibrillation in Blacks versus Whites and the utilization of race in a novel risk score. Am J Cardiol.

[8] Dewland T.A., Olgin J.E., Vittinghoff E., Marcus G.M. (2013). Incident atrial fibrillation among Asians, Hispanics, Blacks, and Whites. Circulation.

[9] Rodriguez C.J., Soliman E.Z., Alonso A. (2015). Atrial fibrillation incidence and risk factors in relation to race-ethnicity and the population-attributable fraction of atrial fibrillation risk factors: the Multi-Ethnic Study of Atherosclerosis. Ann Epidemiol.

[10] Gillott R.G., Willan K., Kain K. (2017). South Asian ethnicity is associated with a lower prevalence of atrial fibrillation despite greater prevalence of established risk factors: a population-based study in Bradford Metropolitan District. Europace.

[11] Wong C.X., Brown A., Tse H.-F. (2017). Epidemiology of atrial fibrillation: the Australian and Asia-Pacific perspective. Heart Lung Circ.

[12] Virani S.S., Alonso A., Aparicio H.J. (2021). Heart disease and stroke statistics—2021 update: a report from the American Heart Association. Circulation.

[13] Huxley R.R., Lopez F.L., Folsom A.R. (2011). Absolute and attributable risks of atrial fibrillation in relation to optimal and borderline risk factors: the Atherosclerosis Risk in Communities (ARIC) Study. Circulation.

[14] Lipworth L., Okafor H., Mumma M.T. (2012). Race-specific impact of atrial fibrillation risk factors in Blacks and Whites in the Southern Community Cohort Study. Am J Cardiol.

[15] Jensen P.N., Thacker E.L., Dublin S., Psaty B.M., Heckbert S.R. (2013). Racial differences in the incidence of and risk factors for atrial fibrillation in older adults: The Cardiovascular Health Study. J Am Geriatr Soc.

[16] O’Neal W.T., Judd S.E., Limdi N.A. (2017). Differential impact of risk factors in Blacks and Whites in the development of atrial fibrillation: the Reasons for Geographic and Racial Differences in Stroke (REGARDS) Study. J Racial Ethn Health Disparities.

[17] O’Neill J., Tayebjee M.H. (2017). Why are South Asians seemingly protected against the development of atrial fibrillation? A review of current evidence. Trends Cardiovasc Med.

[18] Ugowe F.E., Jackson L.R., Thomas K.L. (2018). Racial and ethnic differences in the prevalence, management, and outcomes in patients with atrial fibrillation: a systematic review. Heart Rhythm.

[19] Echocardiographic Normal Ranges Meta-Analysis of the Left Heart Collaboration (2015). Ethnic-specific normative reference values for echocardiographic LA and LV size, LV mass, and systolic function: The EchoNoRMAL Study. JACC Cardiovasc Imaging.

[20] Tsang T.S., Barnes M.E., Bailey K.R. (2001). Left atrial volume: important risk marker of incident atrial fibrillation in 1655 older men and women. Mayo Clin Proc.

[21] Marcus G.M., Olgin J.E., Whooley M. (2010). Racial differences in atrial fibrillation prevalence and left atrial size. Am J Med.

[22] Badertscher P., Gregg D., Baicu C.F. (2021). Racial difference in atrial size and extracellular matrix homeostatic response to hypertension: Is this a potential mechanism of reduced atrial fibrillation in African Americans?. Heart Rhythm O2.

[23] Heckbert S.R., Austin T.R., Jensen P.N. (2020). Differences by race/ethnicity in the prevalence of clinically detected and monitor-detected atrial fibrillation: MESA. Circ Arrhythm Electrophysiol.

[24] Soni A., Karna S., Fahey N. (2019). Age- and-sex stratified prevalence of atrial fibrillation in rural Western India: results of SMART-India, a population-based screening study. Int J Cardiol.

[25] Rooney M.R., Soliman E.Z., Lutsey P.L. (2019). Prevalence and characteristics of subclinical atrial fibrillation in a community-dwelling elderly population: The ARIC Study. Circ Arrhythm Electrophysiol.

[26] Lau C.P., Gbadebo T.D., Connolly S.J. (2013). Ethnic differences in atrial fibrillation identified using implanted cardiac devices. J Cardiovasc Electrophysiol.

[27] Chen M.L., Parikh N.S., Merkler A.E. (2019). Risk of atrial fibrillation in Black versus White Medicare beneficiaries with implanted cardiac devices. J Am Heart Assoc.

[28] Kamel H., Kleindorfer D.O., Bhave P.D. (2016). Rates of atrial fibrillation in Black versus White patients with pacemakers. J Am Heart Assoc.

[29] Tucker N.R., Ellinor P.T. (2014). Emerging directions in the genetics of atrial fibrillation. Circ Res.

[30] Marcus G.M., Alonso A., Peralta C.A. (2010). European ancestry as a risk factor for atrial fibrillation in African Americans. Circulation.

[31] Lubitz S.A., Yin X., Lin H.J. (2017). Genetic risk prediction of atrial fibrillation. Circulation.

[32] Roselli C., Chaffin M.D., Weng L.-C. (2018). Multi-ethnic genome-wide association study for atrial fibrillation. Nat Genet.

[33] Roberts J.D., Hu D., Heckbert S.R. (2016). Genetic investigation into the differential risk of atrial fibrillation among Black and White individuals. JAMA Cardiol.

[34] Liu Y., Ni B., Lin Y. (2015). The rs3807989 G/A polymorphism in CAV1 is associated with the risk of atrial fibrillation in Chinese Han populations. Pacing Clin Electrophysiol.

[35] Chalazan B., Mol D., Sridhar A. (2018). Genetic modulation of atrial fibrillation risk in a Hispanic/Latino cohort. PloS One.

[36] Chalazan B., Mol D., Darbar F.A. (2021). Association of rare genetic variants and early-onset atrial fibrillation in ethnic minority individuals. JAMA Cardiol.

[37] Alzahrani Z., Ornelas-Loredo A., Darbar S.D. (2018). Association between family history and early-onset atrial fibrillation across racial and ethnic groups. JAMA Netw Open.

[38] Gbadebo T.D., Okafor H., Darbar D. (2011). Differential impact of race and risk factors on incidence of atrial fibrillation. Am Heart J.

[39] Golwala H., Jackson L.R., Simon D.N. (2016). Racial/ethnic differences in atrial fibrillation symptoms, treatment patterns, and outcomes: insights from Outcomes Registry for Better Informed Treatment for Atrial Fibrillation Registry. Am Heart J.

[40] Bhave P.D., Lu X., Girotra S., Kamel H., Vaughan Sarrazin M.S. (2015). Race- and sex-related differences in care for patients newly diagnosed with atrial fibrillation. Heart Rhythm.

[41] Eberly L.A., Garg L., Yang L. (2021). Racial/ethnic and socioeconomic disparities in management of incident paroxysmal atrial fibrillation. JAMA Netw Open.

[42] O'Neal W.T., Sandesara P.B., Claxton J.S. (2018). Influence of sociodemographic factors and provider specialty on anticoagulation prescription fill patterns and outcomes in atrial fibrillation. Am J Cardiol.

[43] Mason P.K., Moorman L., Lake D.E. (2010). Gender and racial characteristics of patients referred to a tertiary atrial fibrillation center. J Atr Fibrillation.

[44] Meschia J.F., Merrill P., Soliman E.Z. (2010). Racial disparities in awareness and treatment of atrial fibrillation: the REasons for Geographic and Racial Differences in Stroke (REGARDS) Study. Stroke.

[45] Magnani J.W., Norby F.L., Agarwal S.K. (2016). Racial differences in atrial fibrillation-related cardiovascular disease and mortality: The Atherosclerosis Risk in Communities (ARIC) Study. JAMA Cardiol.

[46] Kabra R., Cram P., Girotra S. (2015). Effect of race on outcomes (stroke and death) in patients > 65 years with atrial fibrillation. Am J Cardiol.

[47] Simpson J.R., Zahuranec D.B., Lisabeth L.D. (2010). Mexican Americans with atrial fibrillation have more recurrent strokes than do non-Hispanic whites. Stroke.

[48] Gu K., Mahtta D., Kaneria A. (2021). Racial disparities among Asian Americans with atrial fibrillation: an analysis from the NCDR® PINNACLE Registry. Int J Cardiol.

[49] Shen A.Y.-J., Contreras R., Sobnosky S. (2010). Racial/ethnic differences in the prevalence of atrial fibrillation among older adults—a cross-sectional study. J Natl Med Assoc.

[50] Patel M.R., Mahaffey K.W., Garg J. (2011). Rivaroxaban versus warfarin in nonvalvular atrial fibrillation. N Engl J Med.

[51] Shen A.Y., Yao J.F., Brar S.S., Jorgensen M.B., Chen W. (2007). Racial/ethnic differences in the risk of intracranial hemorrhage among patients with atrial fibrillation. J Am Coll Cardiol.

[52] Thomas K.L., Piccini J.P., Liang L. (2013). Racial differences in the prevalence and outcomes of atrial fibrillation among patients hospitalized with heart failure. J Am Heart Assoc.

[53] Lewis W.R., Fonarow G.C., Grau-Sepulveda M.V. (2011). Improvement in use of anticoagulation therapy in patients with ischemic stroke: results from Get with the Guidelines—Stroke. Am Heart J.

[54] Birman-Deych E., Radford M.J., Nilasena D.S. (2006). Use and effectiveness of warfarin in Medicare beneficiaries with atrial fibrillation. Stroke.

[55] Yong C., Azarbal F., Abnousi F. (2016). Racial differences in quality of anticoagulation therapy for atrial fibrillation (from the TREAT-AF Study). Am J Cardiol.

[56] Zulkifly H., Cheli P., Lutchman I. (2020). Anticoagulation control in different ethnic groups receiving vitamin K antagonist therapy for stroke prevention in atrial fibrillation. Thromb Res.

[57] Tedla Y.G., Schwartz S.M., Silberman P., Greenland P., Passman R.S. (2020). Racial disparity in the prescription of anticoagulants and risk of stroke and bleeding in atrial fibrillation patients. J Stroke Cerebrovasc Dis.

[58] Haddad A., Bocchese M., Garber R. (2021). Racial and ethnic differences in left atrial appendage occlusion wait time, complications, and periprocedural management. Pacing Clin Electrophysiol.

[59] Steinberg B.A., Holmes D.N., Piccini J.P. (2013). Early adoption of dabigatran and its dosing in US patients with atrial fibrillation: results from the Outcomes Registry for Better Informed Treatment of Atrial Fibrillation. J Am Heart Assoc.

[60] Vaughan Sarrazin M.S., Jones M., Mazur A. (2014). Bleeding rates in Veterans Affairs patients with atrial fibrillation who switch from warfarin to dabigatran. Am J Med.

[61] Sur N.B., Wang K., Di Tullio M.R. (2019). Disparities and temporal trends in the use of anticoagulation in patients with ischemic stroke and atrial fibrillation. Stroke.

[62] Hankey G.J., Stevens S.R., Piccini J.P. (2014). Intracranial hemorrhage among patients with atrial fibrillation anticoagulated with warfarin or rivaroxaban: the rivaroxaban once daily, oral, direct factor Xa inhibition compared with vitamin K antagonism for prevention of stroke and embolism trial in atrial fibrillation. Stroke.

[63] Baik S.H., Hernandez I., Zhang Y. (2016). Evaluating the initiation of novel oral anticoagulants in Medicare beneficiaries. J Manag Care Spec Pharm.

[64] Reddy V.Y., Sievert H., Halperin J. (2014). Percutaneous left atrial appendage closure vs warfarin for atrial fibrillation: a randomized clinical trial. JAMA.

[65] Holmes D.R., Kar S., Price M.J. (2014). Prospective randomized evaluation of the Watchman left atrial appendage closure device in patients with atrial fibrillation versus long-term warfarin therapy: the PREVAIL Trial. J Am Coll Cardiol.

[66] Freeman J.V., Varosy P., Price M.J. (2020). The NCDR Left Atrial Appendage Occlusion Registry. J Am Coll Cardiol.

[67] Vincent L., Grant J., Ebner B. (2021). Racial disparities in the utilization and in-hospital outcomes of percutaneous left atrial appendage closure among patients with atrial fibrillation. Heart Rhythm.

[68] Sparrow R., Sanjoy S., Choi Y.H. (2021). Racial, ethnic and socioeconomic disparities in patients undergoing left atrial appendage closure. Heart.

[69] Khan M.Z., Munir M.B., Darden D. (2021). Racial disparities in in-hospital adverse events among patients with atrial fibrillation implanted with a Watchman left atrial appendage occlusion device: a US national perspective. Circ Arrhythm Electrophysiol.

[70] Badheka A.O., Chothani A., Mehta K. (2015). Utilization and adverse outcomes of percutaneous left atrial appendage closure for stroke prevention in atrial fibrillation in the United States: influence of hospital volume. Circ Arrhythm Electrophysiol.

[71] Ranka S., Acharya P., Sami F.A. (2020). Effect of sex and race on outcomes after left atrial appendage occlusion: a report from national inpatient sample. J Am Coll Cardiol.

[72] Yao X., Gersh B.J., Holmes D.R. (2018). Association of surgical left atrial appendage occlusion with subsequent stroke and mortality among patients undergoing cardiac surgery. JAMA.

[73] Friedman D.J., Piccini J.P., Wang T. (2018). Association between left atrial appendage occlusion and readmission for thromboembolism among patients with atrial fibrillation undergoing concomitant cardiac surgery. JAMA.

[74] Bush D., Martin L.W., Leman R. (2006). Atrial fibrillation among African Americans, Hispanics and Caucasians: clinical features and outcomes from the AFFIRM Trial. J Natl Med Assoc.

[75] Tamariz L., Rodriguez A., Palacio A., Li H., Myerburg R. (2014). Racial disparities in the use of catheter ablation for atrial fibrillation and flutter. Clin Cardiol.

[76] Khalid U., Marzec L.N., Mantini N. (2020). Treatment of AF in American Indians and Alaska Natives: insights from the NCDR PINNACLE-AF Registry. J Am Coll Cardiol.

[77] Hoyt H., Nazarian S., Alhumaid F. (2011). Demographic profile of patients undergoing catheter ablation of atrial fibrillation. J Cardiovasc Electrophysiol.

[78] Naderi S., Rodriguez F. (2014). Wang Y, Foody JM. Racial disparities in hospitalizations, procedural treatments and mortality of patients hospitalized with atrial fibrillation. Ethn Dis.

[79] Hernandez A.F., Fonarow G.C., Liang L. (2007). Sex and racial differences in the use of implantable cardioverter-defibrillators among patients hospitalized with heart failure. JAMA.

[80] Thomas K.L., Al-Khatib S.M., Kelsey R.C. (2007). Racial disparity in the utilization of implantable-cardioverter defibrillators among patients with prior myocardial infarction and an ejection fraction of ≤35%. Am J Cardiol.

[81] Marrouche N.F., Brachmann J., Andresen D. (2018). Catheter ablation for atrial fibrillation with heart failure. N Engl J Med.

[82] Kirchhof P., Camm A.J., Goette A. (2020). Early rhythm-control therapy in patients with atrial fibrillation. N Engl J Med.

[83] Andrade J.G., Champagne J., Deyell M.W. (2018). A randomized clinical trial of early invasive intervention for atrial fibrillation (EARLY-AF)—methods and rationale. Am Heart J.

[84] Shah K.S., Xu H., Matsouaka R.A. (2017). Heart failure with preserved, borderline, and reduced ejection fraction: 5-year outcomes. J Am Coll Cardiol.

[85] Merchant F. (2021). What can administrative data teach us about racial/ethnic disparities in atrial fibrillation management?. JAMA Network Open.

[86] Kummer B.R., Bhave P.D., Merkler A.E. (2015). Demographic differences in catheter ablation after hospital presentation with symptomatic atrial fibrillation. J Am Heart Assoc.

[87] Patel N., Deshmukh A., Thakkar B. (2016). Gender, race, and health insurance status in patients undergoing catheter ablation for atrial fibrillation. Am J Cardiol.

[88] Kwan G.F., Enserro D.M., Benjamin E.J. (2018). Racial differences in hospital death for atrial fibrillation: the National Inpatient Sample 2001-2012. Proclins Cardiol.

[89] Claxton J.S., Lutsey P.L., MacLehose R.F. (2019). Geographic disparities in the incidence of stroke among patients with atrial fibrillation in the United States. J Stroke Cerebrovasc Dis.

[90] Breathett K., Spatz E.S., Kramer D.B. (2021). The groundwater of racial and ethnic disparities research: a statement from *Circulation: Cardiovascular Quality and Outcomes*. Circ Cardiovasc Qual Outcomes.

[91] Egede L.E. (2006). Race, ethnicity, culture, and disparities in health care. J Gen Intern Med.

[92] Kressin N.R., Chapman S.E., Magnani J.W. (2016). A tale of two patients: patient-centered approaches to adherence as a gateway to reducing disparities. Circulation.

[93] United States Agency for Healthcare Quality. National healthcare disparities report 2010. Availabe at: https://www.ahrq.gov/research/findings/nhqrdr/index.html. Accessed November 15, 2021.

[94] Sarraju A., Maron D.J., Rodriguez F. (2020). Under-reporting and under-representation of racial/ethnic minorities in major atrial fibrillation clinical trials. JACC Clin Electrophysiol.

[95] Zhang T., Tsang W., Wijeysundera H.C. (2013). Reporting and representation of ethnic minorities in cardiovascular trials: a systematic review. Am Heart J.

[96] Reddy V.Y., Koruth J., Jais P. (2018). Ablation of atrial fibrillation with pulsed electric fields: an ultra-rapid, tissue-selective modality for cardiac ablation. JACC Clin Electrophysiol.

[97] Bradley C.J., Haines D.E. (2020). Pulsed field ablation for pulmonary vein isolation in the treatment of atrial fibrillation. J Cardiovasc Electrophysiol.

[98] Reddy V.Y., Neuzil P., Koruth J.S. (2019). Pulsed field ablation for pulmonary vein isolation in atrial fibrillation. J Am Coll Cardiol.

[99] Reddy V.Y., Anter E., Rackauskas G. (2020). Lattice-tip focal ablation catheter that toggles between radiofrequency and pulsed field energy to treat atrial fibrillation: a first-in-human trial. Circ Arrhythm Electrophysiol.

[100] Reddy V.Y., Anic A., Koruth J. (2020). Pulsed field ablation in patients with persistent atrial fibrillation. J Am Coll Cardiol.

[101] Mansour M., Calkins H., Osorio J. (2020). Persistent atrial fibrillation ablation with contact force-sensing catheter: The Prospective Multicenter PRECEPT Trial. JACC Clin Electrophysiol.

[102] Bukari A., Nayak H., Aziz Z. (2017). Impact of race and gender on clinical outcomes of catheter ablation in patients with atrial fibrillation. Pacing Clin Electrophysiol.

[103] Frey W.H. America reaches its demographic tipping point. Available at. http://www.brookings.edu/blogs/up-front/posts/2011/08/26-census-race-frey.

[104] Thomas J., Thomas D.J., Pearson T. (1997). Cardiovascular disease in African American and White physicians: the Meharry Cohort and Meharry-Hopkins Cohort Studies. J Health Care Poor Underserved.

[105] Bell M., Lommel T., Fischer J.G. (2009). Improved recognition of heart attack and stroke symptoms after a community-based intervention for older adults, Georgia, 2006-2007. Prev Chronic Dis.

[106] Aronis K.N., Edgar B., Lin W. (2017). Health literacy and atrial fibrillation: relevance and future directions for patient-centred care. Eur Cardiol.

[107] Hall W.J., Chapman M.V., Lee K.M. (2015). Implicit racial/ethnic bias among health care professionals and its influence on health care outcomes: a systematic review. Am J Public Health.

[108] Devine P.G., Forscher P.S., Austin A.J. (2012). Long-term reduction in implicit race bias: a prejudice habit-breaking intervention. J Exp Soc Psychol.

[109] Tony G., Mahzarin B., Brian N. Project Implicit. https://implicit.harvard.edu/implicit/takeatest.html.

[110] Saunders S., Kardia D. Center for Research on Learning and Teaching. Creating inclusive college classrooms. Available at. https://crlt.umich.edu/gsis/p3_1.

[111] LaVeist T.A., Pierre G. (2014). Integrating the 3Ds—social determinants, health disparities, and health-care workforce diversity. Public Health Rep.

[112] Alsan M., Garrick O., Graziani G. (2019). Does diversity matter for health? Experimental evidence from Oakland. Am Econ Rev.

[113] Brown S.D., Lee K., Schoffman D.E. (2012). Minority recruitment into clinical trials: experimental findings and practical implications. Contemp Clin Trials.

[114] Bailey Z.D., Krieger N., Agénor M. (2017). Structural racism and health inequities in the USA: evidence and interventions. Lancet.

[115] Heart Rhythm Society Diversity, equity, inclusion statement. Available at. https://www.hrsonline.org/about-us/structure-and-governance/diversity-equity-inclusion-statement.

